# Assessment of Oral Human Papillomavirus Prevalence in Pediatric and Adult Patients within a Multi-Ethnic Clinic Population

**DOI:** 10.3390/dj10040054

**Published:** 2022-04-01

**Authors:** Melissa Solomon Kornhaber, Taylor Florence, Trexton Davis, Karl Kingsley

**Affiliations:** 1 Department of Advanced Education in Orthodontics and Dentofacial Orthopedics, School of Dental Medicine, University of Nevada, Las Vegas, 1700 W. Charleston Blvd., Las Vegas, NV 89106, USA; kornhabm@unlv.nevada.edu; 2 Department of Clinical Sciences, School of Dental Medicine, University of Nevada, Las Vegas, 1700 W. Charleston Blvd., Las Vegas, NV 89106, USA; floret4@unlv.nevada.edu (T.F.); davist67@unlv.nevada.edu (T.D.); 3 Department of Biomedical Sciences, School of Dental Medicine, University of Nevada, Las Vegas, 1001 Shadow Lane, Las Vegas, NV 89106, USA

**Keywords:** human papillomavirus (HPV), high-risk HPV, oral screening, oral cancer, qPCR screening

## Abstract

Introduction: Human papillomavirus (HPV) encompasses a large family of oncogenic viruses responsible for increasing rates of both cervical and oral cancer, particularly among minority and low-income populations. Although this represents an increasingly significant public health risk, few studies have screened for oral HPV within Nevada. Based upon this information, the primary objective of this study was to provide a temporal analysis of oral HPV screening among a primarily low-income, minority patient population. Methods: This retrospective analysis was reviewed and approved by the Institutional Review Board (IRB). In brief, unstimulated saliva samples were previously obtained from clinical patient volunteers who provided informed consent and pediatric assent (if applicable). DNA was isolated and screened using spectrophotometry for quality (A260:A280 ratio > 1.70) and quantity (concentration > 100 ng). Validated qPCR primers were used to screen repository samples for high-risk HPV strains HPV16 and HPV18. Results: A total of N = 930 samples were identified for this study, which involved n = 555 samples from adults and n = 375 from pediatric patients treated between 2011 and 2019. A demographic analysis revealed nearly equal distribution between males and females with most derived from non-White (minority) patients. A qPCR screening revealed an overall increase in high-risk HPV of 3.17-fold from 5.7% in 2011 to 18.1% in 2019 and a coefficient of determination or R^2^ = 0.764, suggesting a strong, positive correlation between more recent sample years and HPV-positive results, which was observed among both pediatric (R^2^ = 0.671) and adult (R^2^ = 0.971) patients. In addition, although the average age among adult patients increased over time, a significant decrease was observed among pediatric patients from an average of 16.0 years to 14.81 years. Conclusions: These data suggest temporal changes and positive increases in the prevalence of oral HPV among both the pediatric and adult patient samples taken from this clinic population. These data are important as considerations are made regarding which HPV vaccination education and awareness programs are introduced and the specific populations most likely to benefit from these interventions.

## 1. Introduction

The human papillomavirus (HPV) includes a large family of closely related DNA viruses capable of causing cancer in many cells and tissues, including cervical and oral cancers [1,2]. These HPV strains are generally divided into high- and low-risk categories, based upon their propensity to drive oncogenesis in various tissues and organs [3,4,5]. This includes HPV16 and HPV18, which are responsible for the majority of cervical cancers, as well as the vast majority of oral HPV infections worldwide [6,7,8].

Many epidemiologic studies have found the incidence and prevalence of oral cancer may be increasing over time among specific population subgroups, including minorities and low-income patients in both developing and developed countries [9,10,11]. For example, these studies have revealed public health disparities and significantly increased risks in oral cancer among these subgroups in Asia, particularly in developing nations that include India, Pakistan, Bangladesh, and Sri Lanka where public health literacy is low and prevention resources are limited [12]. Although smoking and tobacco or betel quid chewing have been identified as the most prevalent risk factors, an increasing percentage of these cancers have now been confirmed as HPV-positive with growing public health concern about the relatively low levels of HPV vaccination, especially in India [12,13]. In addition, another large developing country with an identified public health problem involving rapidly increasing cases of HPV-positive oral and esophageal cancers is northwestern China, where age-adjusted mortality has reached 150 per 100,000, compared with rates of 2.5 per 100,000 in the United States (U.S.) [12,14].

In other developing areas of the world, such as sub-Saharan Africa, a growing public health concern has been identified with increasing rates and cases of HPV-mediated diseases including cervical and oral cancer—even among more developed countries such as South Africa [15]. In addition, other systematic reviews of HPV infection (both cervical and oral) in areas such as South America, have revealed increasingly high rates among emerging and developing countries with large populations, such as Brazil [16]. These combined data suggest these trends are of particular concern for public health and oral healthcare providers.

Additional epidemiologic studies have found similar results in other developed countries, including many in Europe and the U.S. [17,18]. In particular, developed countries with advanced healthcare systems including Sweden and the U.S. have demonstrated decreased rates of smoking but increasing rates of oral cancer specifically related to HPV16 [17]. This evidence suggests that as many as three-fourths of oral cancers are HPV-positive, which could be prevented with current HPV vaccination [18,19]. According to recent studies, high-risk HPV may be implicated in the majority of oropharyngeal cancers in the U.S., with estimates of incidence increasing by 225% between 1988 and 2004 [18,19,20].

Although these efforts have produced significant data for public health officials to address, few studies have focused more specifically on these subgroups within the population of Nevada [21,22]. For example, a limited number of studies have evaluated the increased risk for some cancers, such as breast and oral cancer in Nevada [23,24,25,26]. However, only a few studies have evaluated the risk and prevalence of oral HPV among these groups in this area [27,28,29]. More studies are needed to increase our understanding of oral HPV prevalence and the oral epidemiology associated with these infections.

Based upon this information, the objective of this study was to determine any changes in prevalence of oral HPV over time among the pediatric and adult patients within a dental school clinic population using an existing repository.

## 2. Study Methods

### 2.1. Study Approval

This retrospective study of existing samples and data was reviewed and approved by the Institutional Review Board (IRB) and the Office for the Protection of Research Subjects (OPRS) at the University of Nevada, Las Vegas (UNLV) under Protocol 1619329-1 titled “Retrospective analysis of Oral Health Status of Dental Population” on 24 July 2020 as Exempt under Federal Regulation 45 CFR 46. The original collection of samples was reviewed and approved by the UNLV IRB under protocol OPRS#880427-1 “The Prevalence of Oral Microbes in Saliva from the University of Nevada Las Vegas (UNLV) School of Dental Medicine (SDM) pediatric and adult clinical population”.

### 2.2. Original Collection Protocol

All samples in the original studies were collected from voluntary participants who were asked to provide informed consent if over the age of 18 years old. For children, both pediatric assent and informed consent from a parent or guardian was required for study participation. Inclusion criteria included patients of record at UNLV-SDM; agreement to voluntary, unpaid study participation; and provision of informed consent and pediatric assent when applicable. Exclusion criteria included any person not a patient of record at UNLV-SDM; any person that did not wish to participate in the voluntary, unpaid study; and any patient that declined to provide either informed consent (over 18 years old) or pediatric assent (under 18 years old).

In brief, patients at the beginning of their clinic visit were provided with sterile collection tubes and asked to provide up to 1.0 mL of unstimulated saliva. Each sample was marked with a randomly generated, non-duplicated identification number to prevent any patient-identifying information from being associated with any specific sample. Basic demographic information was collected at the time of sampling, which included age, sex, race, or ethnicity. No other clinical or demographic information was collected at the time. Each sample was then transferred to a biomedical science laboratory for storage at −20 °C and subsequent processing. Long-term storage was conducted at −80 °C.

### 2.3. DNA Isolation and Quantification

Each sample was thawed and DNA isolated using the GenomicPrep DNA isolation kit from Amersham Biosciences (Buckinghamshire, UK), as previously described [27,28,29]. In brief, equal volumes of the sample (500 µL) were added to Phenol:Chloroform and centrifuged using an Eppendorf 5424 microcentrifuge (Hamburg, Germany) at 10,000× *g* at 4 °C for fifteen minutes to achieve phase separation. The upper (aqueous) phase was transferred to a sterile tube and nucleic acids were precipitated with the addition of an equal volume of isopropanol and centrifuged for ten minutes. The pellet was subsequently washed with molecular grade ethanol and centrifuged for five minutes. The supernatant was aspirated and discarded, and the pellet was resuspended in nuclease-free water. 

Quality and quantity were assessed using a NanoDrop 2000 spectrophotometer from ThermoFisher Scientific (Fair Lawn, NJ, USA) and readings at A260 and A280 nm. The purity (or amount of protein contamination) was determined by the NanoDrop and evaluation of the ratio of absorbance readings at both A260 nm and A280 nm, due to the differences in absorbance between nucleic acids and protein at these wavelengths.

### 2.4. Real-Time qPCR Screening

Samples (N = 930) from pediatric (N = 470) and adult (N = 460) patients that met the minimum acceptable criteria for screening, including purity (A260:A280 ratio > 1.70) and concentration (>[100 ng/uL]), were screened in triplicate using validated primers for both oral high-risk strains of HPV (HPV16, HPV18) using Glyceraldehyde-3-Phosphate Dehydrogenase or GAPDH as the positive qPCR control, as previously described [27,28,29]. In brief, each reaction was performed using the ABsolute SYBR green mix from ThermoFisher Scientific, which consisted of 2x ABsolute SYBR green master mix (12.5 µL), Forward primer at 10 µM (1.5 µL), Reverse primer at 10 µM (1.5 µL), DNA sample (1.0 ng), and up to 8.0 µL of nuclease-free water. Each reaction was run for 15 min at 95 °C for enzyme activation, followed by 40 cycles of denaturation for 15 s at 95 °C, annealing at the primer-specific temperature for 30 s and extension at 72 °C for an additional 30 s.

Glyceraldehyde 3-phosphate dehydrogenase (GAPDH)
GAPDH forward:
5′-ATCTTCCAGGAGCGAGATCC-3′; 20 nt, 55% GC, Tm: 66 °C
GAPDH reverse:
5′-ACCACTGACACGTTGGCAGT-3′; 20 nt, 55% GC, Tm: 70 °C
Optimal PCR Tm: 61 °C
HPV16
Forward primer:
5′-ATGTTTCAGGACCCACAGGA-3′; 20 nt; 50% GC: Tm = 66 °C
Reverse primer:
5′-CCTCACGTCGCAGTAACTGT-3′; 20 nt; 55% Tm = 67 °C
Optimal PCR Tm: 65 °C
HPV18
Forward primer:
5′-ATGGCGCGCTTTGAGGATCC-3′; 20 nt; 60% GC: Tm = 71 °C
Reverse primer:
5′-GCATGCGGTATACTGTCTCT-3′; 20 nt; 50% GC: Tm = 64 °C
Optimal PCR Tm: 63 °C

### 2.5. Statistical Analysis

Demographic information regarding patient sex, age, and race/ethnicity as self-reported (White or Minority, with subgroups matching the clinic patient intake forms listed as Hispanic, Black, Asian, and Other) were compiled in Microsoft Excel (Redmond, WA, USA). Descriptive statistics for overall age average and range, percentage of males and females, as well as racial and ethnic categories were compiled. Comparisons between the study sample and the overall clinic demographics were made using a Chi square (χ2) analysis and reported with degrees of freedom (d.f.) and the resulting p-value (using α = 0.05 level of significance), which is appropriate for categorical, non-parametric data. Two-tailed *t*-tests were used to determine any differences between the average age of study samples and the overall age of the adult or pediatric clinic populations, which is appropriate for a continuous, parametric data analysis.

Data regarding HPV screening (HPV positive, HPV negative) and high-risk HPV strains (HPV16, HPV18) were compiled and presented as descriptive statistics for each year (2011–2019). The coefficient of determination or R^2^ was used to assess the significance and strength of correlation between the sampling year and the presence or absence of HPV-positive samples (weak positive correlation: 0–0.299, moderate positive correlation 0.3–0.699, strong positive correlation 0.7–1.0).

## 3. Results

This retrospective study involved the retrieval and study of N = 930 pediatric (N = 470) and adult (N = 460) patient samples (Table 1). More specifically, the percentage of study samples from adult males and females was nearly equal (48.5%, 51.5%, respectively), which was not significantly different from the percentage of males and females from the overall clinical population, *p* = 0.1290. However, the percentage of adult samples from racial and ethnic minorities was significantly different with lower percentages of minorities (59.3%), and Hispanics in particular (23.4%), than in the clinic population (65.4%, 58.6%, respectively), *p* = 0.001. In addition, the proportion of study samples from White (40.7%), Black (19.5%), and Asian (16.4%) adults was higher than has been observed in the clinic population (34.6%, 10.2%, and 6.6%, respectively). The average sample age for adults was 42.08 years (range 18–88 yrs.), which was not significantly younger than the average age for adult clinic patients of 42.31 years (range 18–89 yrs.), *p* = 0.411.

The analysis of pediatric samples revealed the percentage of pediatric males and females was nearly equal (46.4%, 53.6%, respectively), which was not significantly different from the overall percentage of males and females from the pediatric clinical population, *p* = 0.6123. In addition, the percentage of pediatric samples from racial and ethnic minorities was not significantly different between the study sample (78.4%) and the pediatric clinic population (75.3%), *p* = 0.4884. However, a more in-depth analysis revealed a lower percentage of Hispanics in the study sample (31.5%), and higher percentages of Blacks (27.2%) and Asians (20%) compared with the pediatric clinic population (52.1%, 11.8%, and 11.4%, respectively). The average sample age for pediatric patients was 13.03 years (range 5–17 yrs.), which was significantly different than the average overall age for pediatric clinic patients of 10.44 years (range 0–17 yrs.), *p* = 0.019.

To determine if the samples had sufficient quantity and quality for a qPCR analysis, all samples were screened using a spectrophotometric analysis (Table 2). These data demonstrated that all pediatric samples had sufficient concentrations of DNA (average 218.4 ng/uL) and purity (average A260:A280 = 1.74) for the subsequent qPCR screening. In addition, adult samples also demonstrated sufficient concentrations of DNA (average 334.1 ng/uL) and purity (average A260:A280 = 1.76) for further processing and screening.

**Figure 1:**
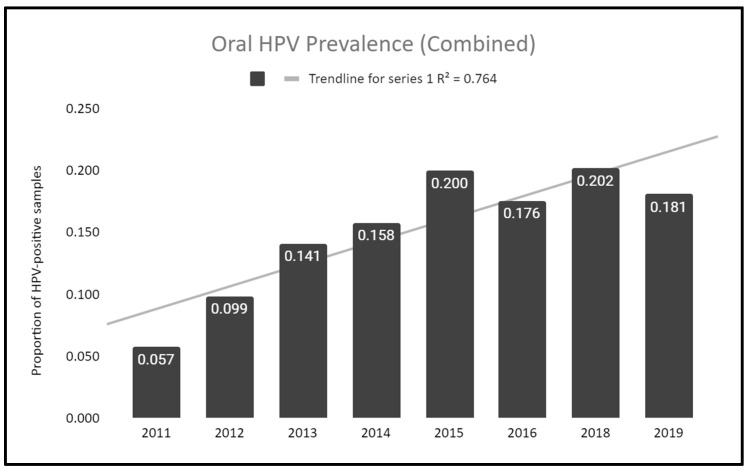
Change in prevalence of high-risk oral HPV over time. Analysis of both pediatric and adult patients revealed an increase in high-risk HPV of 3.17-fold from 5.7% in 2011 to 18.1% in 2019, with the coefficient of determination or R^2^ = 0.764, suggesting a strong, positive correlation between more recent sample years and HPV-positive results.

To determine if this population experienced any changes in prevalence of oral HPV over time, HPV screening results for the study sample, including both pediatric and adult patients, were analyzed and graphed (Figure 1). More specifically, this analysis demonstrates that the proportion of samples testing positive for high-risk HPV increased from 5.7% in 2011 to 18.1% in 2019, an increase of 3.17-fold over this time period. A more in-depth analysis revealed the coefficient of determination or R^2^ = 0.764, which suggests a positive correlation between the more recent sample years and HPV-positivity. 

Raw data for HPV-positive and HPV-negatives samples from this screening were compiled (Table 3). These data demonstrate an average sample number of approximately 116 per year, ranging between n = 65 (2015) and n = 229 (2018) for the lowest and highest, respectively. The total number of HPV-positive samples was n = 133, and HPV-negative samples was n = 806, with a total sample number of N = 930.

**Figure 2:**
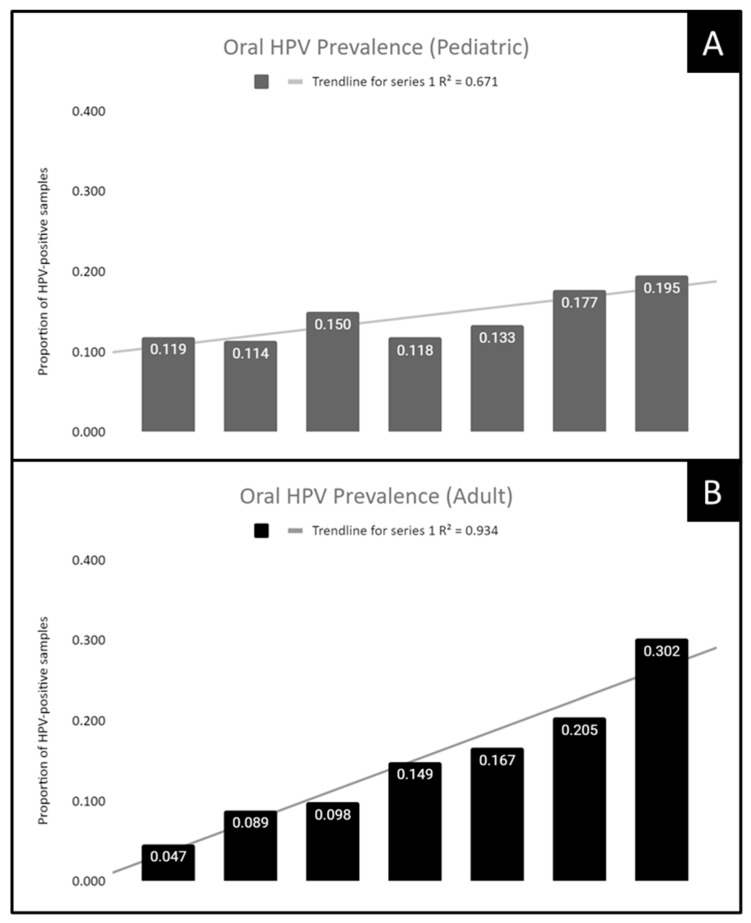
Prevalence of high-risk oral HPV increased over time among both pediatric and adult samples. (**A**) Analysis of pediatric samples revealed an increase of 61.5% between 2012 (11.9%) and 2019 (19.5%) with a coefficient of determination or R^2^ = 0.671. (**B**) Evaluation of adult samples revealed an increase of 6.42-fold between 2011 (4.7%) and 2018 (30.2%) with R^2^ = 0.934.

To assess whether the temporal association between sampling year and proportion of HPV-positive samples was restricted to adult samples, pediatric samples, or found within both populations, these data were analyzed separately (Figure 2). For example, an analysis of the pediatric sample data revealed an increase of 61.5% between 2012 (11.9%) and 2019 (19.5%). An analysis of the coefficient of determination or R^2^ = 0.671, which suggests this population subgroup exhibits a positive correlation between the more recent sample years and HPV-positivity (Figure 2A). In addition, an analysis of the adult sample data revealed an increase of 6.42-fold between 2011 (4.7%) and 2018 (30.2%). The coefficient of determination or R^2^ = 0.934, which suggests a very high correlation between the year of sampling and proportion of HPV-positive samples (Figure 2B).

**Figure 3:**
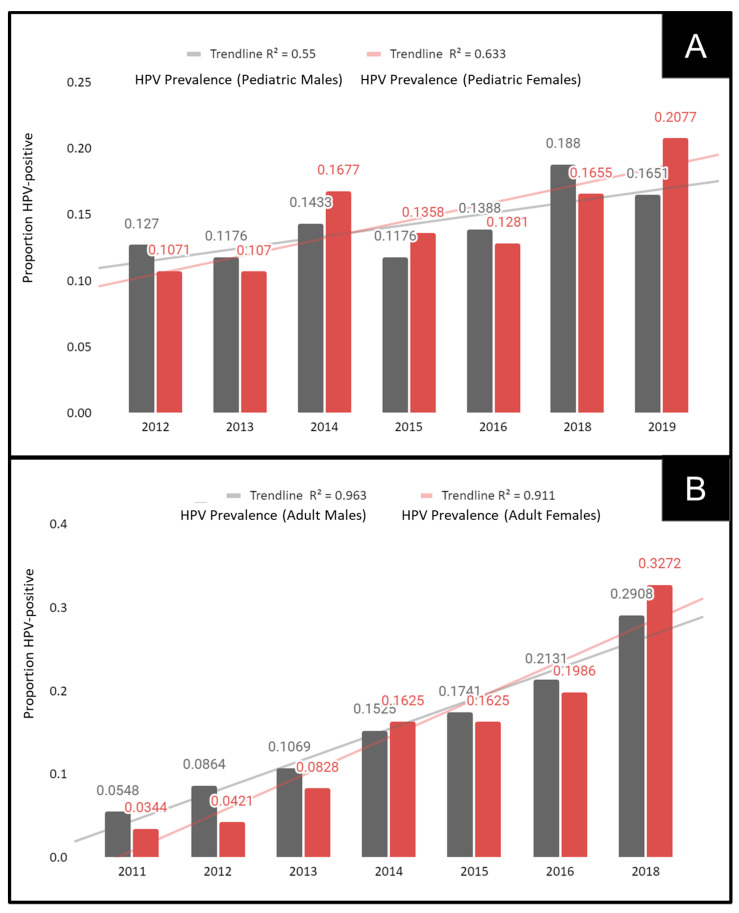
Analysis of oral HPV prevalence by sex. (**A**) Prevalence of oral HPV significantly increased among pediatric males (40.4%) between 2012 and 2019, *p* = 0.0018, with higher increases (93.9%) observed among pediatric females over the same interval, *p* = 0.00013. (**B**) Oral HPV among adult males increased more than 5.3-fold, *p* = 0.0001, between 2011 and 2018, with more than 9.5-fold increases observed among adult females during the same time period, *p* = 0.00001.

To determine if these observations of increasing oral HPV prevalence were more closely associated with males or females, data from the pediatric and adult samples were sorted, analyzed, and graphed (Figure 3). This analysis demonstrated that increases in oral HPV were found among both male and female pediatric patient samples, including the significant 40.4% increase observed among males between 2012 (12.7%) and 2019 (16.5%), *p* = 0.0018. A more significant increase of 93.9% was observed among pediatric females between 2012 (10.7%) and 2019 (20.77%), *p* = 0.00013 (Figure 3A).

The analysis of adult male and female patient samples also revealed significant and dramatic increases in oral HPV (Figure 3B). In brief, the increase in oral HPV among adult males increased from 5.5% in 2011 to 29.1% in 2018, i.e., an increase of more than 5.3-fold, *p* = 0.0001. In addition, the increase among adult females increased from 3.4% in 2011 to 32.72% in 2018, i.e., an increase of more than 9.5-fold, *p* = 0.00001.

**Figure 4:**
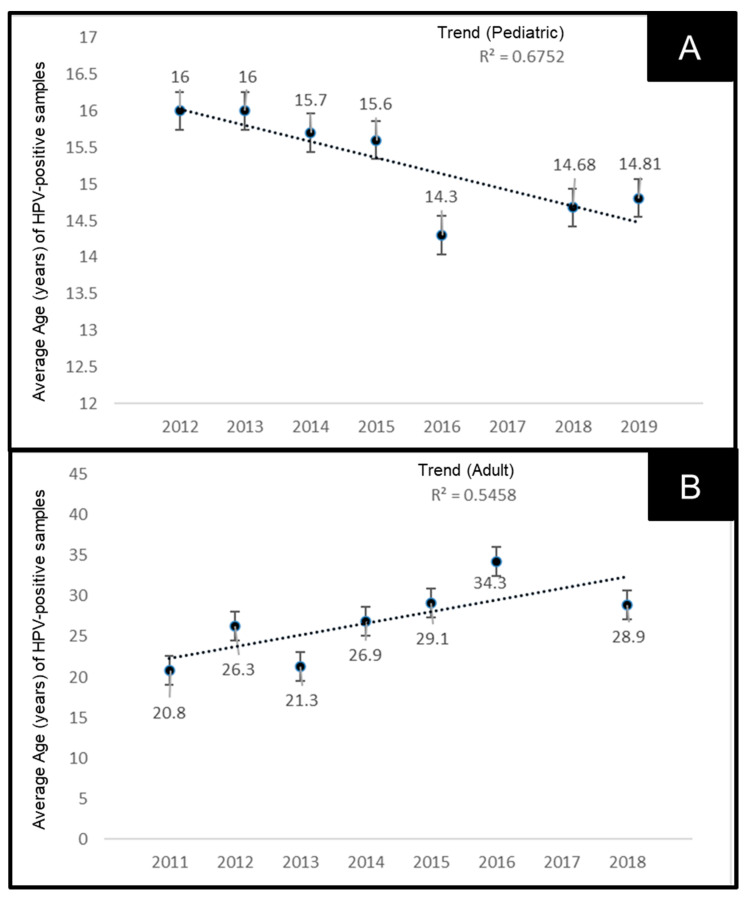
Analysis of average age of HPV-positive samples. (**A**) Average age of pediatric patients with oral HPV decreased from 16 years in 2012 to 14.81 in 2019, R^2^ = 0.6752. (**B**) Average age of adult patients with oral HPV has increased from 20.8 years in 2011 to 28.9 years in 2018, R^2^ = 0.5458.

Due to the increased prevalence of oral HPV observed among both pediatric and adult patient samples, more detailed analyses were performed to determine if there were any changes in the average age of these patients (Figure 4). This analysis demonstrates that the average age of pediatric patients with oral HPV has decreased significantly from an average age of 16 years in 2012 to 14.81 in 2019, R^2^ = 0.6752 (Figure 4A). However, the average age of adult patients testing positive for oral HPV has increased significantly from an average of 20.8 years in 2011 to 28.9 years in 2018, R^2^ = 0.5458 (Figure 4B).

To more accurately assess the changes in average age among the HPV-positive samples, the average ages were compiled along with the age range from both pediatric and adult samples (Table 4). These data demonstrated that not only has the average age decreased among pediatric samples over time (16.0 years in 2012 to 14.81 years in 2019), but the age range has also extended into lower ages from the more narrow range of 15–17 years in 2012, to 12–17 years between 2016 and 2018. In contrast, the average age among adult samples has increased over time from 20.8 years in 2011 to 28.9 years in 2018, which has also included an increase in the age range from 18–26 years in 2011 to 18–49 years in 2018.

## 4. Discussion

The primary goal of this study was to determine if there were any changes in the prevalence of oral HPV over time using an existing dental school-based saliva repository. This study successfully identified nearly 1000 samples collected over the past decade from both adult and pediatric patients, which were successfully screened for the most commonly identified high-risk HPV strains: HPV16 and HPV18. These results revealed a positive increase over time in the percentage of samples harboring oral HPV between 2011 and 2019—observations that were consistent among both pediatric and adult populations. These results support similar recent observations that demonstrate significant and persistent increases in oral HPV infection with high-risk strains, such as HPV16 and HPV18 [30,31].

These results provide support for other recent clinical observations, adding to the growing scientific consensus that more information is needed to accurately determine the burden of oral HPV infection among healthy populations [32,33]. Moreover, as vaccination controversy and hesitancy appear to have increased with the SARS-CoV-2 pandemic, and routine healthcare and vaccinations have been delayed or postponed, more information regarding the incidence and prevalence of oral HPV infection (especially among adolescents and youth) may be needed to understand the scope of the problem at hand and potential costs of delay or inaction [34,35]. As the potential public health and prevention benefits of early HPV vaccination of adolescents and young adults have now become evident, concerted research efforts will be needed to provide clear and accurate information about the increasing presence of oral HPV infection and the potential impact of vaccine hesitancy and postponement, especially among pediatric patients [36,37,38].

Healthcare providers, including dentists and orthodontists, are rapidly becoming an increasingly important part of public health efforts to increase vaccine awareness and knowledge, as well as providing up-to-date information to counter misinformation and reduce beliefs in vaccine myths [39,40,41]. As part of this effort, researchers from this group and others have recently sought to determine knowledge gaps, awareness, and beliefs among dental students and residents to evaluate potential gaps and address vaccine concerns and hesitancy [42,43]. These studies support a growing body of research that demonstrates dentists and other oral healthcare providers are, in fact, willing to discuss HPV vaccination and participate in vaccine administration if they are given sufficient information about the increasing prevalence and risks of oral HPV infection, particularly among adolescent and young adult populations [44,45,46]. Given that some dental specialists, such as orthodontists, regularly visit with the at-risk groups identified in this study, including both pediatric (12–17 years old) and adult (18–24 years old) patients, novel pathways for increasing vaccine knowledge and awareness and countering misinformation may be developed to coincide with their frequent patient interactions [47,48].

The results from this study provide critical information regarding temporal trends observed in both pediatric and adult oral HPV infection over time, which is significant due the fact that only three previous oral HPV studies have been conducted in this patient population—all of which were cross-sectional, single time point screenings conducted between five and ten years ago [27,28,29]. In fact, these observations regarding increases in oral HPV infection support other recent observations regarding worsening trends in both oral health and cancer epidemiology in Nevada that might be explained, in part, by these results [49,50]. Furthermore, as the age of oral HPV infection among pediatric patients appears to be decreasing over time within this patient population, the importance of early intervention and pediatric vaccination becomes more evident.

Although this study provides novel insights into this patient population, there are some limitations to this study that must also be considered. First, this was a retrospective study of previously collected saliva samples and is therefore cross-sectional in nature. No information is available to determine how or when the patients became HPV-positive and whether this oral infection was eventually cleared. Next, although each previous saliva-based study attempted to reduce bias in sample collection, there was some evidence to suggest the over sampling of minorities among the adult patient population. Future studies of this nature might include more rigorous plans for randomized patient selection, which might reduce bias in the collection and subsequent analysis of patient samples. Finally, this study does not delve into the reasons why there may be increasing prevalence among younger and younger pediatric patients; however, some recent evidence has suggested that increases in vaccine hesitancy and unfounded beliefs about general vaccine side effects and vaccine safety concerns have recently reduced overall and HPV-specific vaccination rates [51,52,53,54,55].

## 5. Conclusions

Based upon this information, the results of this study have revealed the presence of temporal changes and an increased prevalence of oral HPV infection among both the pediatric and adult patients over time. These data provide critical epidemiologic information that must be considered as new guidelines and recommendations are made regarding which HPV vaccination education and awareness programs are introduced to healthcare providers (including dentists) and the specific populations, such as pediatric patients, that are most likely to benefit from these interventions.

## Figures and Tables

**Table 1:** Study sample demographics.

Demographics	Study Sample	Clinic	Statistical Analysis
**Sex**			
Adult—Males	N = 269 (48.5%)	50.9%	χ2 = 2.305, d.f. = 1 *p* = 0.1290
Adult—Females	N = 286 (51.5%)	49.1%	
**Race/Ethnicity**			
White	N = 226 (40.7%)	34.6%	χ2 = 16.444, d.f. = 1 *p* = 0.0001
Minority	N = 329 (59.3%)	65.4%	
Hispanic	N = 130 (23.4%)	58.6%	
Black	N = 108 (19.5%)	10.2%	
Asian/Other	N = 91 (16.4%)	6.6%	
**Age**			
Average/Range Median	42.80 yrs. 42 yrs.	42.31 yrs. 41 yrs.	Two-tailed *t*-test *p* = 0.411
Range	18–88 yrs.	18–89 yrs.	
**Sex**			
Pediatric—Males	N = 174 (46.4%)	47.2%	χ2 = 0.257, d.f. = 1 *p* = 0.6123
Pediatric—Females	N = 201 (53.6%)	52.8%	
**Race/Ethnicity**			
White	N = 81 (21.6%)	24.7%	χ2 = 0.480, d.f. = 1 *p* = 0.4884
Minority	N = 294 (78.4%)	75.3%	
Hispanic	N = 118 (31.5%)	52.1%	
Black	N = 102 (27.2%)	11.8%	
Asian/Other	N = 75 (20.0%)	11.4%	
**Age**			
Average/Range Median	13.03 yrs. 12 yrs.	10.44 yrs. 10 yrs.	Two-tailed *t*-test *p* = *0.019*
Range	5–17 yrs.	0–17 yrs.	

**Table 2:** Analysis of study sample DNA.

Study Sample	DNA Concentration	DNA Purity (A260:A80)
Pediatric samples N = 470	Average: 218.4 ng/uL ± 88.2 Range: 110–631.1 ng/uL	Average: 1.74 Range: 1.70–1.79
Adult samples N = 460	Average: 334.1 ng/uL ± 91.1 Range: 135–815.1 ng/uL	Average: 1.76 Range: 1.70–1.81

**Table 3:** qPCR HPV screening by year.

Year	HPV-Positive Samples	HPV-Negative Samples	Total Sample Number	Proportion of HPV-Positives
2011	n = 5	n = 82	n = 87	0.057
2012	n = 15	n = 137	n = 152	0.099
2013	n = 11	n = 67	n = 78	0.141
2014	n = 21	n = 112	n = 133	0.158
2015	n = 13	n = 52	n = 65	0.200
2016	n = 13	n = 74	n = 87	0.176
2018	n = 40	n = 199	n = 229	0.202
2019	n = 15	n = 83	n = 98	0.181
**Total**	**n = 133**	**n = 806**	**N = 930**	

**Table 4:** Age summary of HPV-positive samples.

Year	Pediatric	Adult
2011	N/A	Average: 20.8 years Range (18–26 years)
2012	Average: 16.0 years Range (15–17 years)	Average: 26.25 years Range (18–55 years)
2013	Average: 16.0 years Range (15–17 years)	Average: 21.3 years Range (18–27 years)
2014	Average: 15.7 years Range (14–17 years)	Average: 26.9 years Range (18–41 years)
2015	Average: 15.6 years Range (13–17 years)	Average: 29.1 years Range (18–66 years)
2016	Average: 14.3 years Range (12–16 years)	Average: 34.3 years Range (23–52 years)
2018	Average: 14.68 years Range (13–17 years)	Average: 28.9 years Range (18–49 years)
2019	Average: 14.81 years Range (14–17 years)	N/A

## Data Availability

The data presented in this study are available upon request from the corresponding author. The data are not publicly available due to the study protocol data protection parameters requested by the IRB and OPRS for the initial study approval.

## Abbreviations

Human papillomavirus (HPV); Institutional Review Board (IRB); Deoxyribonucleic acid (DNA); United States (U.S.); Office for the Protection of Research Subjects (OPRS); University of Nevada, Las Vegas (UNLV); School of Dental Medicine (SDM); Relative Centrifugal Force (RCF); nanograms (ng); nucleotides (nt); quantitative polymerase chain reaction (qPCR); melting temperature (Tm); years (yrs.)

## References

[1] Brianti P., De Flammineis E., Mercuri S.R. (2017). Review of HPV-related diseases and cancers. New Microbiol..

[2] Jiang S., Dong Y. (2017). Human papillomavirus and oral squamous cell carcinoma: A review of HPV-positive oral squamous cell carcinoma and possible strategies for future. Curr. Probl. Cancer.

[3] Egawa N., Doorbar J. (2017). The low-risk papillomaviruses. Virus Res..

[4] Stanley M. (2010). Pathology and epidemiology of HPV infection in females. Gynecol. Oncol..

[5] Yusupov A., Popovsky D., Mahmood L., Kim A.S., Akman A.E., Yuan H. (2019). The nonavalent vaccine: A review of high-risk HPVs and a plea to the CDC. Am. J. Stem Cells.

[6] De Sanjosé S., Brotons M., Pavon M.A. (2018). The natural history of human papillomavirus infection. Best Pract. Res. Clin. Obstet. Gynaecol..

[7] Doorbar J., Egawa N., Griffin H., Kranjec C., Murakami I. (2015). Human papillomavirus molecular biology and disease association. Rev. Med. Virol..

[8] Sabatini M.E., Chiocca S. (2019). Human papillomavirus as a driver of head and neck cancers. Br. J. Cancer.

[9] Yete S., D’Souza W., Saranath D. (2018). High-Risk Human Papillomavirus in Oral Cancer: Clinical Implications. Oncology.

[10] Timbang M.R., Sim M.W., Bewley A.F., Farwell D.G., Mantravadi A., Moore M.G. (2019). HPV-related oropharyngeal cancer: A review on burden of the disease and opportunities for prevention and early detection. Hum. Vaccines Immunother..

[11] Simonidesová S., Hamšíková E., Klozar J., Tachezy R. (2019). The prevalence of oral HPV infection in healthy populations: A systematic review with a focus on European populations. Epidemiol. Mikrobiol. Imunol..

[12] Rao S.V.K., Mejia G., Roberts-Thomson K., Logan R. (2013). Epidemiology of Oral Cancer in Asia in the Past Decade—An Update (2000–2012). Asian Pac. J. Cancer Prev..

[13] Aswathy S., Reshma J., Avani D. (2015). Epidemiology of cervical cancer with special focus on India. Int. J. Women’s Health.

[14] Zheng S., Vuitton L., Sheyhidin I., Vuitton D.A., Zhang Y., Lu X. (2010). Northwestern China: A place to learn more on oesophageal cancer. Part one: Behavioural and environmental risk factors. Eur. J. Gastroenterol. Hepatol..

[15] Williamson A.-L., Passmore J.-A., Rybicki E., Marais D., Rybicki E. (2002). Human Papillomavirus (HPV) Infection in Southern Africa: Prevalence, Immunity, and Vaccine Prospects. IUBMB Life.

[16] Colpani V., Falcetta F.S., Bidinotto A.B., Kops N.L., Falavigna M., Hammes L.S., Benzaken A.S., Maranhão A.G.K., Domingues C.M.A.S., Wendland E.M. (2020). Prevalence of human papillomavirus (HPV) in Brazil: A systematic review and meta-analysis. PLoS ONE.

[17] Nasman A., Du J., Dalianis T. (2019). A global epidemic increase of an HPV induced tonsil and tongue-base cancer-potential benefit from a pan-gender use of HPV vaccine. J. Intern. Med..

[18] Moore K.A., Mehta V. (2015). The Growing Epidemic of HPV-Positive Oropharyngeal Carcinoma: A Clinical Review for Primary Care Providers. J. Am. Board Fam. Med..

[19] Cleveland J.L., Junger M.L., Saraiya M., Markowitz L.E., Dunne E.F., Epstein J.B. (2011). The connection between human papillomavirus and oropharyngeal squamous cell carcinomas in the United States. J. Am. Dent. Assoc..

[20] Chaturvedi A.K., Engels E.A., Pfeiffer R.M., Hernandez B.Y., Xiao W., Kim E., Jiang B., Goodman M.T., Sibug-Saber M., Cozen W. (2011). Human Papillomavirus and Rising Oropharyngeal Cancer Incidence in the United States. J. Clin. Oncol..

[21] Pharr J.R., Kachen A., Cross C. (2019). Health Disparities Among Sexual Gender Minority Women in the United States: A Population-Based Study. J. Community Health.

[22] Pabayo R., Cook D.M., Harling G., Gunawan A., Rosenquist N.A., Muennig P., Pabayo R., Cook D.M., Harling G., Gunawan A. (2019). State-level income inequality and mortality among infants born in the United States 2007–2010: A Cohort Study. BMC Public Health.

[23] Kingsley K., O’Malley S., Ditmyer M., Chino M. (2008). Analysis of oral cancer epidemiology in the US reveals state-specific trends: Implications for oral cancer prevention. BMC Public Health.

[24] Bunnell A., Pettit N., Reddout N., Sharma K., O’Malley S., Chino M., Kingsley K. (2010). Analysis of primary risk factors for oral cancer from select US states with increasing rates. Tob. Induc. Dis..

[25] Callahan K.E., Pinheiro P.S., Cvijetic N., Kelly R.E., Ponce C.P., Kobetz E.N. (2016). Worse Breast Cancer Outcomes for Southern Nevadans, Filipina and Black Women. J. Immigr. Minor. Health.

[26] Siegel R.L., Fedewa S.A., Miller K.D., Goding-Sauer A., Pinheiro P.S., Martinez-Tyson D., Jemal A. (2015). Cancer statistics for Hispanics/Latinos, 2015. CA Cancer J. Clin..

[27] O’Turner D., Williams-Cocks S.J., Bullen R., Catmull J., Falk J., Martin D., Mauer J., Barber A.E., Wang R.C., Gerstenberger S.L. (2011). High-risk human papillomavirus (HPV) screening and detection in healthy patient saliva samples: A pilot study. BMC Oral Health.

[28] Flake C., Arafa J., Hall A., Ence E., Howard K., Kingsley K. (2012). Screening and detection of human papillomavirus (HPV) high-risk strains HPV16 and HPV18 in saliva samples from subjects under 18 years old in Nevada: A pilot study. BMC Oral Health.

[29] Tiku V., Todd C.J., Kingsley K. (2016). Assessment of Oral Human Papillomavirus Prevalence in a Multi-ethnic Pediatric Clinic Population. Compend. Contin. Educ. Dent..

[30] Brouwer A.F., Campredon L.P., Walline H.M., Marinelli B.M., Goudsmit C.M., Thomas T.B., Delinger R.L., Lau Y.K., Andrus E.C., Nair T. (2022). Incidence and clearance of oral and cervicogenital HPV infection: Longitudinal analysis of the MHOC cohort study. BMJ Open.

[31] Giuliani E., Rollo F., Donà M.G., Garbuglia A.R. (2021). Human Papillomavirus Oral Infection: Review of Methodological Aspects and Epidemiology. Pathogens.

[32] Morais E., Kothari S., Roberts C., Yen G., Chen Y.-T., Lynam M., Pedrós M., Mirghani H., Alemany L., Pavon M.A. (2021). Oral human papillomavirus (HPV) and associated factors among healthy populations: The design of the PROGRESS (PRevalence of Oral hpv infection, a Global aSSessment) study. Contemp. Clin. Trials.

[33] Zhang Y., D’Souza G., Fakhry C., O’Bigelow E., Usyk M., Burk R.D., Zhao N. (2022). Oral HPV associated with differences in oral microbiota beta diversity and microbiota abundance. J. Infect. Dis..

[34] Morán-Torres A., Pazos-Salazar N.G., Téllez-Lorenzo S., Jiménez-Lima R., Lizano M., Reyes-Hernández D.O., Marin-Aquino J.D.J., Manzo-Merino J. (2021). HPV oral and oropharynx infection dynamics in young population. Braz. J. Microbiol..

[35] Löffler P. (2021). Review: Vaccine Myth-Buster—Cleaning Up With Prejudices and Dangerous Misinformation. Front. Immunol..

[36] Kaczmarczyk K.H., Yusuf H. (2021). The impact of HPV vaccination on the prevention of oropharyngeal cancer: A scoping review. Community Dent. Health.

[37] McClure C.C., Cataldi J.R., O’Leary S.T. (2017). Vaccine Hesitancy: Where We Are and Where We Are Going. Clin. Ther..

[38] Stahl J.-P., Cohen R., Denis F., Gaudelus J., Martinot A., Lery T., Lepetit H. (2016). The impact of the web and social networks on vaccination. New challenges and opportunities offered to fight against vaccine hesitancy. Med. Mal. Infect..

[39] Mammas I.N., Dalianis T., Doukas S.G., Zaravinos A., Achtsidis V., Thiagarajan P., Theodoridou M., Spandidos D.A. (2019). Paediatric virology and human papillomaviruses: An update. Exp. Ther. Med..

[40] Dean T.C., Gilliland A.E., Cameron J.E. (2020). Parents’ receptiveness to oral health clinic-based vaccination. Vaccine.

[41] Alawi F. (2020). Oral health care providers should be administering vaccines. Oral Surg. Oral Med. Oral Pathol. Oral Radiol..

[42] Geoghegan S., O’Callaghan K.P., Offit P.A. (2020). Vaccine Safety: Myths and Misinformation. Front. Microbiol..

[43] Mann S.K., Kingsley K. (2020). Human Papillomavirus (HPV) Vaccine Knowledge, Awareness and Acceptance among Dental Students and Post-Graduate Dental Residents. Dent. J..

[44] Rutkoski H., Tay D.L., Dixon B.L., Pinzon L.M., Mooney R., Winkler J.R., Kepka D. (2020). A Multi-state Evaluation of Oral Health Students’ Knowledge of Human Papillomavirus-Related Oropharyngeal Cancer and HPV Vaccination. J. Cancer Educ..

[45] Kepka D., Rutkoski H., Pappas L., Tay D.L., Winkler J.R., Dixon B., Velazquez A., Pinzon L.M. (2019). US oral health students’ willingness to train and administer the HPV vaccine in dental practices. Prev. Med. Rep..

[46] Harris K.L., Tay D., Kaiser D., Praag A., Rutkoski H., Dixon B.L., Pinzon L.M., Winkler J.R., Kepka D. (2019). The perspectives, barriers, and willingness of Utah dentists to engage in human papillomavirus (HPV) vaccine practices. Hum. Vaccines Immunother..

[47] Walker K.K., Jackson R.D., Sommariva S., Neelamegam M., Desch J. (2019). USA dental health providers’ role in HPV vaccine communication and HPV-OPC protection: A systematic review. Hum. Vaccines Immunother..

[48] Daley E.M., Thompson E.L., Beckstead J., Driscoll A., Vamos C., Piepenbrink R.P., Desch J., Merrell L., Cayama M.B.R., Owens H. (2021). Discussing HPV and oropharyngeal cancer in dental settings: Gender and provider-type matter. Hum. Vaccines Immunother..

[49] Murphy C.C., Yang Y.C. (2018). Use of Age-Period-Cohort Analysis in Cancer Epidemiology Research. Curr. Epidemiol. Rep..

[50] Foote K., Foote D., Kingsley K. (2021). Surveillance of the Incidence and Mortality of Oral and Pharyngeal, Esophageal, and Lung Cancer in Nevada: Potential Implications of the Nevada Indoor Clean Air Act. Int. J. Environ. Res. Public Health.

[51] La Fauci V., Squeri R., Genovese C., Anzalone C., Fedele F., Squeri A., Alessi V. (2019). An observational study of university students of healthcare area: Knowledge, attitudes and behaviour towards vaccinations. Clin. Ter..

[52] Marti M., de Cola M., Macdonald N.E., Dumolard L., Duclos P. (2017). Assessments of global drivers of vaccine hesitancy in 2014—Looking beyond safety concerns. PLoS ONE.

[53] Lane S., MacDonald N.E., Marti M., Dumolard L. (2018). Vaccine hesitancy around the globe: Analysis of three years of WHO/UNICEF Joint Reporting Form data-2015–2017. Vaccine.

[54] Kulkarni S., Harvey B., Prybylski D., Jalloh M.F. (2021). Trends in classifying vaccine hesitancy reasons reported in the WHO/UNICEF Joint Reporting Form, 2014–2017: Use and comparability of the Vaccine Hesitancy Matrix. Hum. Vaccines Immunother..

[55] Zhang J., Xue H., Calabrese C., Chen H., Dang J.H.T. (2021). Understanding Human Papillomavirus Vaccine Promotions and Hesitancy in Northern California through Examining Public Facebook Pages and Groups. Front. Digit. Health.

